# Low reproducibility of equivocal categories of the Bethesda System for Reporting Thyroid Cytology makes the associated risk of malignancy specific to the diagnostic center

**DOI:** 10.1007/s12020-021-02781-3

**Published:** 2021-06-12

**Authors:** Dorota Słowińska-Klencka, Mariusz Klencki, Joanna Duda-Szymańska, Jarosław Szwalski, Bożena Popowicz

**Affiliations:** 1 Department of Morphometry of Endocrine Glands, Chair of Endocrinology, Medical University of Lodz, 251 Pomorska Str, 92-213 Lodz, Poland; 2 Department of Pathomorphology, Chair of Oncology, Medical University of Lodz, 251 Pomorska Str, 92-213 Lodz, Poland

**Keywords:** Thyroid cancer, FNA, Bethesda system, Reproducibility, Cytology

## Abstract

**Purpose:**

Equivocal categories (III, IV, V) of the Bethesda System for Reporting Thyroid Cytology (BSRTC) are characterized by high variability of the estimated risk of malignancy. The aim of the study was to analyze the reproducibility of classification of nodules into an equivocal category and the frequency of malignancy (FoM) observed in such categories.

**Methods:**

Five experienced cytopathologists from three centers (A, B, C) independently performed reclassification of smears obtained from 213 thyroid nodules with equivocal routine cytology and known results of the postoperative histopathological examination.

**Results:**

The interobserver agreement among all cytopathologists was poor, with a Krippendorff’s alpha coefficient equaling 0.34. The intra-center agreement was higher than the inter-center (fair vs poor). Pathologists of the center A classified smears into categories II and III significantly less often and categories IV and V more often than pathologists of centers B and C. The joint FoM of nodules classified into any of categories IV–VI (regarded as an indication for surgery) was different among centers (A: 40.0%, B: 66.7%, C: 80.6%). The FoM of category III nodules with features of nuclear atypia (AUS) in center B and C was two times higher than that of other nodules of category III (FLUS), while in center A the FoM was similar.

**Conclusions:**

The use of published data on the risk of malignancy in nodules of particular BSRTC categories without concern for the uniqueness of the diagnostic center may lead to erroneous conclusions.

## Introduction

The diagnostics of thyroid nodules is challenging. The basic diagnostic method in use is the fine-needle aspiration biopsy (FNA), but unfortunately up to 30% of FNA outcomes is classified into one of three equivocal categories (III, IV, or V) according to the Bethesda System for Reporting Thyroid Cytology (BSRTC) [1, 2]. An additional difficulty is that the risk of malignancy (RoM) of nodules classified into the same equivocal category is highly variable [3, 4].

The category V—the suspicion of malignancy (SM)—is commonly regarded as an indication for surgical treatment because of the high RoM: amounting to 45–75%, according to the authors of the BSRTC (45–60% if the diagnosis of non-invasive follicular thyroid neoplasm with papillary-like nuclear features -NIFTP is regarded as a benign lesion and 50–75% if it is regarded as a malignancy), and even reaching 90%, according to some reports [1–4].

The category IV—the suspicion of follicular neoplasm (SFN)/suspicion of Hürthle cell tumor (SHT)—is related to the lower RoM (10–40 or 25–40% depending on the interpretation of NIFTP as a benign or malignant lesion, respectively), according to the authors of the BSRTC, but it is also regarded as an indication for thyroid surgery [1–4]. In such cases, diagnostic uncertainties about nodule’s malignancy are usually dispelled only by the postoperative histopathological examination. A certain preoperative diagnosis is often not possible even if molecular tests were performed [4, 5]. In the original BSRTC, cases that demonstrated the nuclear features of papillary carcinoma (PTC) were excluded from the category IV [1]. Currently, follicular-patterned cases with mild nuclear changes can also be classified into the category IV as long as true papillae and intranuclear pseudoinclusions are absent [2].

The category III: follicular lesion of undetermined significance (FLUS)/atypia of undetermined significance (AUS) is related to the most diverse RoM [2, 3, 6–12]. It embraces specimens in which the cytomorphological findings are not representative of a benign lesion, yet the degree of cellular, nuclear and/or architectural atypia is not sufficient to render a diagnosis of SFN/SHCT or SM. Initially, the category III was meant to include no more than 7% of FNA results, and its RoM was to remain under 15% [1]. Consequently, the usual management was to consist of the repeated FNA with the consideration of molecular testing if possible. Over time it was found that the frequency of category III was significantly higher in some centers and its RoM differed according to the nature of the atypia and it could even reach above 70%, particularly in the case of nodules with nuclear atypia, commonly referred to as AUS [2, 3, 6–11].

The above-mentioned large variability of the RoM related to particular equivocal categories of the BSRTC may be a result of factors independent of the diagnostic center, e.g., iodine supply (in iodine-deficient areas the RoM of nodules is lower because of the high incidence of non-neoplastic nodules). But that variability may also be caused by center-specific factors, such as different interpretation of the rules for the classification of nodules into BSRTC categories. Thus, the aim of our study was the assessment of the reproducibility of the classification of nodules into the equivocal category of the BSRTC and the analysis of the frequency of malignancy (FoM) observed in particular categories when several experienced pathologists independently reevaluated the same set of smears.

## Materials and methods

The analysis comprised smears obtained from 213 thyroid nodules with equivocal outcome of routine FNA (categories III, IV, or V of the BSRTC: 127, 53, and 33 cases, respectively) and the known result of the postoperative histopathological examination. Biopsies were performed in 213 patients (mean age: 54.2 ± 15.6), including 189 (88.7%) women and 24 (11.3%) men, in years 2010–2018 in a single diagnostic center (denoted throughout the text with the letter B). All patients gave their informed consent.

FNAs were performed following regular procedures on thyroid nodules with a diameter of at least 5 mm (and usually over 1 cm) and at least one malignancy risk factor (US or clinical). In all cases, two aspirations of a nodule were done. Smears were fixed with 95% ethanol solution and stained with hematoxylin and eosin (H&E). The results of FNA were formulated according to the Bethesda classification in the version prior to the modification in 2017. Patients with a cytological outcome of SFN/SHT or SM were routinely referred for surgical treatment. In the case of the diagnosis of FLUS/AUS, the surgical treatment was performed based on the patient’s preference or due to the large size of the goiter or the presence of other clinical risk features. The histopathologic examination was performed according to the standard procedure and its results were formulated according to the WHO classification of thyroid tumors that was in effect at the time. The reclassification of the histopathological examination in order to reveal cases of NIFTP was not performed.

The postoperative histopathological examination of the biopsied nodules revealed 67 (30.5%) malignant neoplasms, including a single case of follicular tumor with uncertain malignant potential (FT-UMP) and a single case of NIFTP, as well as 146 (68.5%) benign lesions. Among cancers there were 41 (61.2%) PTC, 12 (17.9%) follicular carcinomas (FTC), 8 (11.9%) Hürthle cell carcinomas (HTC), 3 (4.5%) medullary carcinomas, and 1 (1.5%) anaplastic carcinoma. The benign nodules comprised 91 (62.3%) cases of non-neoplastic nodular goiter, 41 (28.1%) follicular thyroid adenomas and 14 (9.6%) Hürthle cell adenomas.

### Analyses, statistical evaluation

The outcomes of thyroid cytological examinations performed by five pathologists were compared. Pathologists belonged to three different diagnostic centers (labeled as A, B, or C), the center A: pathologists A1 and A2, center B: pathologists B1 and B2, center C: pathologist C1. All participating cytopathologists had at least 15 years’ experience in thyroid cytopathology and assessed about 1500–3500 FNAs a year (only one pathologist in the center C had a comparable experience in thyroid cytology—25 years’ one with about 3500 FNAs evaluated yearly). The H&E is a routine staining of thyroid biopsy specimens in all participating centers. All the centers serve as a tertiary thyroid referral center. The center A is a part of the Academic Center for Oncology. The center B is an academic pathology center providing pathomorphological services to several academic hospitals with various profiles and to two large endocrine outpatient clinics. The center C is a large, non-academic center providing cytologic and histopathologic diagnostic services for other medical centers. Cytopathologists were informed that all evaluated smears had been classified into the categories III–V of the BSRTC during the routine diagnostics but they did not know the exact category nor the result of postoperative histopathological examination. The pathologists were instructed to apply the BSRTC classification in the modification of 2017. They were asked to identify follicular-patterned cases with mild nuclear changes among nodules classified into the category IV, as there was a possibility of the follicular variant of PTC or NIFTP in such nodules. Those cases were denoted by FP-NC. Mild nuclear changes were defined as the increased nuclear size, nuclear contour irregularity, and/or chromatin clearing. The pathologists were also asked to identify smears with nuclear atypia among the biopsies classified into the category III. In such cases, PTC cannot be excluded and those smears were denoted by AUS. They were defined as smears with the presence of any abnormal nuclear features such as nuclear enlargement, grooves, prominent nucleoli, abnormal chromatin pattern, alteration of nuclear contour and shape, and/or presence of intranuclear cytoplasmic inclusions in an otherwise predominantly benign-appearing sample. In all three centers pathologists used only one term, “FLUS” to denote category III in routine diagnostics until 2018. Recently, following the national guidelines and the practice common in numerous publications, they began to describe the cases of category III presenting nuclear atypia with the term “AUS”. The cytopathologists were given all relevant clinical information such as the patient’s age, thyroid function status, diagnosis of autoimmune thyroid disease or any malignant tumor and treatment applied.

The distribution of FNA outcomes between particular categories was assessed for each pathologist. The distribution was analyzed for the whole group of nodules and separately for benign and malignant nodules, including the most frequent carcinomas (PTC and FTC). The number and percentage of cases characterized by the complete agreement of diagnoses (in terms of the BSRTC category) between the five pathologists were determined and the coefficient of concordant diagnoses was calculated. Similar analyzes were performed for all possible pairs of participating pathologists with a particular concern for the agreement of diagnoses within and between the diagnostic centers. Then the agreement in diagnosing the following equivocal cytological categories: III (and separately AUS), IV (and separately FP-NC), and V was assessed within the diagnostic centers. For this purpose, the number of concordant diagnoses of each category was divided by the number of all diagnoses of that category at the center. Finally, the FoM was compared for all nodules classified into each equivocal BSTRC category by each pathologist. We did not use the term ‘RoM’ because the nodules were not randomly included into the study. Those comparisons also included AUS and FP-NC subcategories. For the sake of statistical analysis, the FT-UMP and NIFTP diagnoses were regarded as a “malignancy”. In our opinion, as well as the opinion of authors of the Bethesda classification, such an approach is more clinically relevant because tumors of both these types should be treated surgically [2].

The study design was approved by the Local Bioethics Committee at the National Research Institute of Oncology - as a part of the studies carried out for the realization of the grant MILESTONE. The approval code is “13/2015/1/2016”.

The statistical analysis was performed with Dell Statistica (data analysis software system), version 13, Dell Inc. (2016), Round Rock, TX, USA. The comparison of frequency distributions was performed with chi2 test (with modifications appropriate for the number of analyzed cases). The reports of the cytopathologists were evaluated for the interobserver variability by calculating the percentage of agreement. Statistically, the degree of reproducibility in the formulating of equivocal BSRTC categories was estimated with Krippendorff’s alpha coefficient jointly among all pathologists and for all possible pairs of them [13]. The value of alpha coefficient was calculated in two variants: with the Bethesda classification regarded as a nominal scale or as an ordinal scale. Such an approach was adopted because of reasonable doubts about the ordinal nature of that classification, with a number of reports showing the RoM associated with the category III as higher than the category IV, and the category I higher than the category II [3, 4]. Krippendorff’s alpha coefficient was interpreted using the following criteria: 0–0.4 poor agreement; 0.41–0.75 fair agreement; 0.76–1.0 almost perfect agreement. The value of 0.05 was assumed as the level of significance.

## Results

### Comparison of the distribution of diagnoses between BSRTC categories

**Fig. 1:**
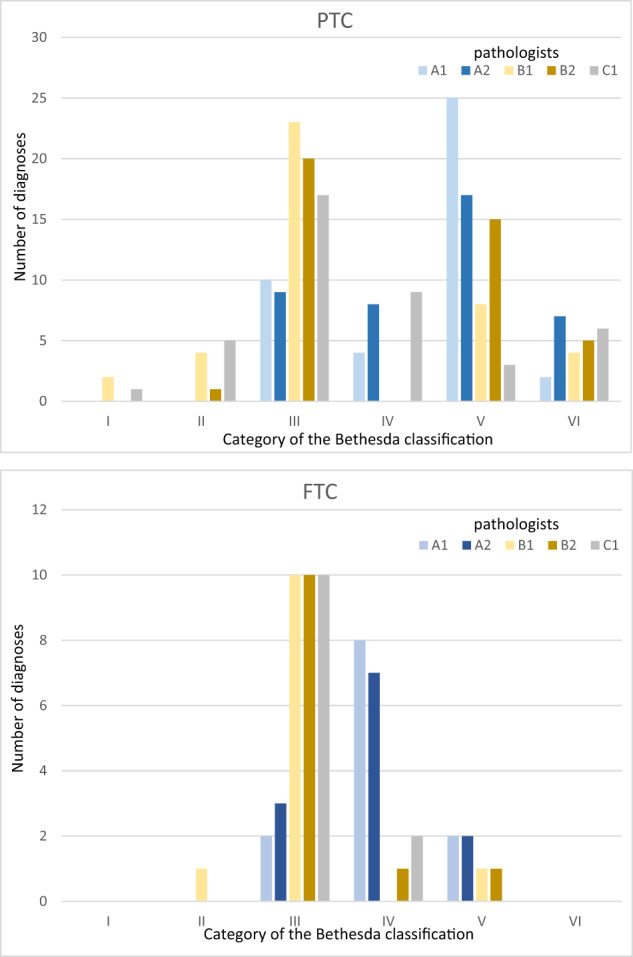
The distribution of FNA outcome categories in the group of the most frequent malignant neoplasms (PTC and FTC) for individual pathologists. PTC papillary carcinoma, FTC follicular carcinoma

Table 1 shows the distribution of FNA outcomes formulated by each pathologist among BSRTC categories. In the whole examined group of nodules, pathologists of the center A classified smears into categories II and III significantly less often and categories IV and V more often than pathologists of other centers. In the case of category III that difference was twofold, and in the case of categories II, IV, and V even severalfold. A similar pattern was observed when malignant neoplasms and benign nodules were analyzed separately. Pathologist C1 formulated the diagnosis of category V conspicuously rarely, assigning only 6% of cancers to that category, while pathologist A1 assigned 53.7% of cancers. However, pathologist C1 assigned only 0.7% of benign nodules to that category, while pathologist A1—17.8%. Pathologists of the center A classified most PTCs into category V (pathologist A1: 61%, pathologist A2: 41.4%), and most FTCs into category IV (A1: 66.7%, A2: 58.3%). Pathologists of centers B and C classified most PTCs as well as FTCs into category III (PTC, B1: 56.1%, B2: 48.8%, C1: 41.4%; FTC, all: 83.3%) (see Fig. 1). Supplementary Tables S1-S3 (Online Resource 1) show detailed information on the distribution of diagnoses by all pathologists for nodules assigned to one of equivocal BSRTC categories by at least one of them.

**Table 1:** The distribution of cytological diagnoses formulated by particular pathologists among categories of the Bethesda classification

Pathologist	Category of the Bethesda classification—percentage (number)					
	I	II	III	IV	V	VI
	All nodules					
A1	0.5 (1) *<0.05 vs B1*	0.5 (1) *<0.0001 vs B1,B2* <*0.001 vs C1*	31.9 (68) *<0.0001 vs B1,B2,C1*	37.1 (79) *<0.0001 vs B1,B2,C1*	29.1 (62) *<0.0001 vs B1,B2,C1* <*0.01 vs A2*	0.9 (2) *<0.05 vs A2*
A2	0.5 (1) <0.05 *vs B1*	2.8 (6) *<0.001 vs B2* <*0.05 vs* *B1,C1*	35.7 (76) *<0.0001 vs B1,B2,C1*	38.0 (81) *<0.0001 vs B1,B2,C1*	18.3 (39) *<0.0001 vs C1* <*0.001 vs B1*	4.7 (10)
B1	3.8 (8)	9.9 (21)	73.7 (157) *<0.05 vs B2*	4.2 (9) *<0.01 vs B2*	6.6 (14)	1.9 (4)
B2	0.9 (2)	8.0 (17)	63.8 (136)	12.7 (27)	11.7 (25)	2.8 (6)
C1	2.3 (5)	11.7 (25)	71.4 (152)	8.5 (18)	2.3 (5) *<0.001 vs B1* <*0.05 vs B2*	3.8 (8)

	Malignant neoplasms					
A1	–	–	17.9 (12) *<0.0001 vs B1,B2,C1*	25.4 (17) *<0.001 vs B1* <*0.01 vs B2*	53.7 (36)^a,b^ *<0.0001 vs B1,C1* <*0.05 vs A2,B2*	3.0 (2)
A2	–	–	22.4 (15) *<0.0001 vs B1* <*0.001 vs B2,C1*	29.9 (20) *<0.001 vs B1,B2*	35.8 (24)^b^ *<0.0001 vs C1* <*0.05 vs B1*	11.9 (8)^a^
B1	3.0 (1)	7.5 (5)	62.7 (42)^a,b^	3.0 (2)	17.9 (12)	6.0 (4)
B2	–	1.5 (1)	55.2 (37)^b^	6.0 (4)	29.8 (20)^a^	7.5 (5)
C1	1.5 (1)	7.5 (5)	53.7 (36)^a,b^	19.4 (13) *<0.05 vs B1,B2*	6.0 (4) *<0.001 vs B2*	11.9 (8)

	Benign lesions					
A1	0.7 (1)	0.7 (1) *<0.001 vs B1,B2,C1*	38.4 (56) *<0.0001 vs B1,B2,C1*	42.5 (62) *<0.0001 vs B1,B2,C1*	17.8 (26) *<0.0001 vs B1,B2,C1*	–
A2	0.7 (1)	4.1 (6) *<0.05 vs B1,B2,C1*	41.8 (61) *<0.0001 vs B1,B2,C1*	41.8 (61) *<0.0001 vs B1,B2,C1*	10.3 (15) *<0.05 vs B1,B2,C1*	1.4 (2)
B1	4.1 (6)	11.0 (16)	78.8 (115)	4.8 (7)	1.4 (2)	–
B2	1.4 (2)	11.0 (16)	67.8 (99) *<0.05 vs B1,C1*	15.8 (23) *<0.01 vs B1,C1*	3.4 (5)	0.7 (1)
C1	2.7 (4)	13.7 (20)	79.5 (116)	3.4 (5)	0.7 (1)	–

Among cancers there was:

^a^1 case of non-invasive follicular thyroid neoplasm with papillary-like nuclear features (NIFTP)

^b^1 case of follicular tumor with uncertain malignant potential (FT-UMP)

Italic values indicates statistical significance.

### Analysis of the agreement between diagnoses

Full agreement of cytological diagnoses among all five pathologists was found in only 31 cases (14.6%): single cases of category I, II, and VI, two cases of category IV and V each, and 24 cases of category III. There were five malignant neoplasms among those 31 cases (7.5% of all cancers, four PTC and one FTC) and 26 (17.8%) benign lesions. The concordance of diagnoses was lower for malignant neoplasms than benign nodules (see Table 2). In the whole examined group of nodules the interobserver agreement among all the cytopathologists was poor, with a Krippendorff’s alpha coefficient equaling to 0.20 when the Bethesda classification was regarded as a nominal scale, and 0.34 when it was regarded as an ordinal scale. When the agreement of diagnoses was analyzed within particular centers, higher values of Krippendorff’s alpha coefficient that showed fair agreement were observed: in the center A—0.50 and 0.60 and in the center B—0.44 and 0.53, respectively. In the center A concordant categorization between two pathologists was observed in 65.7% of cases, and in the center B—71.8% of cases (Table 2). In the center A, where the frequencies of categories III and IV were similar, the agreement of diagnoses of category III was lower than category IV: 15.0% (19/127) vs 56.6% (59/108), *p* < 0.0001 and lower than category V: 44.3% (31/70), *p* < 0.0001. In the center B, where category III dominated other equivocal categories, the agreement of diagnoses of that category was higher than categories IV and V: 69.2% (119/172) vs. 33.3% (9/27) and vs. 34.5% (10/29), respectively (*p* < 0.0005 in both cases). The percentages of the agreement of diagnoses of AUS in category III and FP-NC in category IV were under 50% in both centers—AUS, center A: 36.6% (26/71), center B: 31.9% (15/47); FP-NC, center A: 26.3% (10/38), center B: 44.4% (4/9).

**Table 2:** The number and percentage of FNA in which there was an agreement in the assigned category of the Bethesda classification between particular pathologists

Pathologist	Percentage (number) of concurring diagnoses			
	A1	A2	B1	B2
	**All cases—213**			
A2	65.7 (140)			
B1	32.9 (70)	37.6 (80)		
B2	45.5 (97)	48.4 (103)	71.8 (153)	
C1	29.6 (63)	32.4 (69)	73.7 (157)	58.2 (124)
	**Malignant neoplasms—67**			
A2	62.7 (42)			
B1	26.9 (19)	29.9 (20)		
B2	40.3 (28)	40.3 (27)	65.7 (44)	
C1	19.4 (13)	28.4 (19)	61.2 (41)	44.8 (30)
	**Benign nodules—146**			
A2	67.1 (98)			
B1	35.6 (51)	41.1 (60)		
B2	48.6 (69)	52.1 (76)	74.7 (109)	
C1	34.2 (50)	34.2 (50)	79.5 (116)	64.6 (94)

The agreement between particular pathologists of the centers A and B ranged from 32.9 to 48.4%. Pathologist C1 showed agreement of diagnoses with pathologists of the center B (58.2–73.7%) that was twofold higher than with the center A (29.6–32.4%) (see Table 2). When the concordance of diagnoses of two pathologists of different centers was analyzed, fair agreement was found only for a single pair of them: B1 and C1, with the value of Krippendorff’s alpha coefficient equal to 0.43 (with the Bethesda classification regarded as a nominal scale) or 0.59 (an ordinal scale). That agreement concerned mainly category III, which was most frequently formulated— 73.6% (131/178). In the case of other equivocal categories the concordance of their diagnoses was low—12.5% in category IV (3/24), and 26.7% in category V (4/15), *p* < 0.0005 vs. category III in both cases. In the case of other pairs of pathologists of different centers the agreement of diagnoses was poor (Table 3).

**Table 3:** The agreement of diagnoses between pathologists as measured with the use of Krippendorff’s alpha coefficient and the assumption of ordinal nature of the Bethesda classification

Pathologist	Krippendorff’s alpha coefficient			
	A1	A2	B1	B2
A2	0.60			
B1	0.03	0.14		
B2	0.26	0.35	0.53	
C1	0.01	0.15	0.59	0.37

### Analysis of FoM in nodules categorized into the equivocal BSTRC category

Table 4 shows the comparison of the FoM in nodules categorized into the equivocal BSTRC category by particular pathologists. The lowest differences in the FoMs were observed in category III, but a detailed analysis of AUS subcategory revealed significant discrepancies (Table 5). In the center A, category III was dominated by AUS, especially in the case of pathologist A1 (AUS: 76.5%). The FoM in AUS subcategory did not exceed 20% and was similar to other nodules of category III for both pathologists of the center A. In the centers B and C, AUS subcategory comprised less than 25% of diagnoses of category III. For pathologists B1 and C1 AUS nodules had a twofold higher FoM than other nodules of category III (pathologist B1: 44.1 vs. 22.0%, pathologist C1: 42.9 vs. 17.9%, respectively, *p* < 0.01 in both cases), lower differences were observed for pathologist B2 (36.7 vs. 24.5%, NS). Nodules diagnosed as AUS were more often PTC than other nodules of category III for all pathologists, but those differences were insignificant (A1: 88.9 vs. 66.7%, A2: 66.7 vs. 50.0%, B1: 73.3 vs. 50.0%, B2: 63.6 vs. 50.0%, C1: 60.0 vs. 38.1%, respectively).

**Table 4:** Number and percentage of cancers among all nodules classified into particular equivocal categories of the Bethesda classification

Pathologist	Equivocal categories [No./% of cancers]		
	III	IV	V
A1	12/17.6	17/21.5	36/58.1
A2	15/19.7	20/24.7	24/61.5
B1	42/26.8	2/22.2	12/85.7
B2	37/27.2	4/14.8	20/80.0
C1	36/23.7	13/72.2^ab^	4/80.0

^a^*p* < 0.0001 vs. A1, A2, B2

^b^*p* < 0.05 vs. B1

**Table 5:** Number and percentage of AUS cases within category III of the Bethesda system and of FP-NC cases within category IV and the data on the FoM in these nodules

Pathologist	Category III				Category IV			
	AUS		Others		FP-NC		Others	
	No./% of cases	FoM	No./% of cases	FoM	No./% of cases	FoM	No./% of cases	FoM
A1	52/76.5^ab^	17.3^c^	16/23.5^ab^	18.8	16/20.3^d^	18.8	63/79.7^d^	22.2
A2	45/59.2^a^	20.0^c^	31/40.8^a^	19.4	32/39.5	31.3	49/60.5	20.4
B1	34/21.7	44.1	123/78.3	22.0	6/66.7	16.7	3/33.3	33.3
B2	30/22.1	36.7	106/77.9	24.5	7/25.9^e^	14.3	20/74.1^e^	15.0
C1	35/23.0	42.9	117/77.0	17.9	11/61.1	90.9^fg^	7/38.9	42.9

*AUS* atypia of undetermined significance, *FoM* frequency of malignancy, *FP-NC* follicular-patterned cases with mild nuclear changes

^a^*p* < 0.0001 vs. B1, B2, C1

^b^*p* < 0.05 vs. A2

^c^*p* < 0.01 vs. B1, C1

^d^*p* < 0.01 vs. A2, B1, C1

^e^*p* < 0.05 vs. C1

^f^*p* < 0.001 vs. A1, A2, B2

^g^*p* < 0.05 vs. B1

In the case of category IV, there were also differences in the frequency of FP-NC diagnoses. Pathologists A1 and B2 identified FP-NC two times less often than pathologists B1 and C1 (Table 5). But only in the case of pathologist C1 the FoM in FP-NC nodules, or nodules of category IV in general, was significantly higher (with a more than three-fold difference in both cases) than for other pathologists (Tables 4 and 5). The diagnoses of category IV by pathologist C1 also showed the highest percentage of PTC among cancers (pathologist C1: 84.6% PTC vs. A1: 23.5%, A2: 40.0%, B1 and B2: 0.0%, *p* < 0.05 in all cases except for pathologist B1). The same was true for FP-NC subcategory (pathologist C1: 90.0% PTC vs. A1, B1, B2: 0.0% and A2: 50.0%, NS). No significant differences were found in the FoM between nodules diagnosed as FP-NC and other nodules of category IV for any pathologist.

Among nodules of category V, the FoM was 20 percentage points higher in the case of pathologists of centers B and C than pathologists of the center A, but those differences did not reach the significance threshold (Table 4).

The joint FoM of nodules classified into any of categories IV-VI (regarded as an indication for the surgical treatment) was the highest in the case of pathologist C1—80.6% (25/31), and for other pathologists, it was as follows: B1—66.7% (18/27), B2—50.0% (28/58), A2—40.0% (52/130) and A1—38.5% (55/143) (*p* < 0.0001 C1 vs. A1 and A2; *p* < 0.005 C1 vs. B2; *p* < 0.05 B1 vs. A1 and A2).

## Discussion

The introduction of the Bethesda classification for thyroid cytological diagnoses was an important step toward better preoperative diagnostics of thyroid nodules. The primary aim of the classification was to make the rules for the categorization of the smears as well as the nomenclature uniform. That aim has been achieved to a high extent, mainly because nowadays the classification is commonly used worldwide. Unfortunately, not all expectations have been met. In particular, the RoM of nodules in each equivocal category has not been successfully established [3, 4]. This problem is well illustrated by our study in which experienced cytopathologists, specialized in diagnostics of the thyroid gland, reevaluated over 200 thyroid biopsies classified into an equivocal category in the routine examination. We found that during the reevaluation most of the smears were again classified into the equivocal category, but their distribution among particular BSRTC categories differed significantly between the pathologists. That was the case in spite of the fact that the pathologists followed the same guidelines and had similar long-time experience in the thyroid cytopathology. The latter was probably the reason why the pathologists did not feel bound by the original diagnosis; as experts they were used to the consultation of difficult cases and verification of the primary diagnosis. The analysis of the incidence of particular BSRTC categories showed that pathologists of the centers B and C were more willing to assign smears into categories II and III, while those of the center A had more readiness to use categories IV and V, as well as AUS subcategory of category III. Those differences in the frequency of BSRTC categories were as high as severalfold and were observed in both cancers and benign nodules. That had a significant impact on the differences in the frequency of revealing cancers in nodules classified by particular pathologists into the same BSRTC category. Pathologists of the center A, who were more inclined to use categories of a potentially higher RoM, obtained the lower FoM in those categories than other pathologists. It was probably a consequence of specific characteristics of the center A, which is an oncology center while the others are not. In consequence, pathologists of center A relatively rarely evaluate smears obtained from non-neoplastic lesions, where features of atypia may result from chronic thyroiditis, hyperthyroidism, antithyroid agent use, or radioiodine treatment. On the other hand, they are more vigilant about features of atypia and tend to classify cases with the slightest degree of atypia to higher BSRTC categories. In particular, they are more eager to use categories that are an indication for surgical treatment (IV–VI). Among patients with nodules, which pathologists of the center A classified into such a category, only 40.0% actually had a cancer, while of the center B—66.7%, and the center C—80.6%. Obviously, these percentages do not reflect the joint RoM of three equivocal diagnostic categories in particular centers. But they show how big the differences may be in the FoM in the case of biopsy with an equivocal microscopic image. Taking that into account, endocrinologists need to know how to interpret particular categories of FNA outcomes in the context of the diagnostic center where the examination was made. Only then is it possible to weigh potential benefits and risks related to the surgery in a patient with equivocal and alerting cytological outcome correctly, especially when significant comorbidities occur.

We expected to find poor agreement in diagnosing category III, as its definition is the least precise. That problem was indicated by other investigators [6–11], and even the authors of the Bethesda classification [1, 2]. Accordingly, in the center A where pathologists assigned smears into each of three equivocal categories with a similar frequency, the reproducibility of category III was markedly lower than categories IV or V. Interestingly, the FoM in nodules classified as AUS by pathologists of center A was similar to that in nodules without features of nuclear atypia (usually denoted as FLUS), which is concordant with the intentions of the authors of the BSRTC classification. On the other hand, the FoM among nodules diagnosed as AUS by other pathologists was twice as high as in other nodules of category III (without nuclear atypia) which was concordant with the majority of reports on that matter. It is probably a consequence of the same factors that led to earlier discussed differences in FoM of nodules of categories IV–VI between these centers (lower threshold of nuclear atypia intensity when categorizing smears into the AUS subgroup in the center A). Because the estimated RoM for nodules with nuclear atypia in some centers approaches the RoM of nodules in category V, the decision about the surgical treatment is made without repeating FNA, particularly if the nodule shows a highly suspicious sonographic pattern [11, 14, 15]. However, it is hard to formulate decisive recommendations in this area with such large differences between diagnostic centers. In our sample, the FoM in nodules assigned to category V in centers B and C was two times higher than in AUS nodules and four times higher than in FLUS nodules. But in center A the FoM in nodules of category V was 20% lower than in center B and three times higher than in nodules diagnosed as AUS or FLUS that showed similar frequencies of malignancy, as it was already mentioned. That problem could probably be solved to some degree by the wider use of molecular tests in patients with equivocal cytology. Unfortunately their use is limited by high costs and the lack of satisfactory validation in various populations. ATA guidelines recommend the use of such tests in nodules with equivocal cytology, especially of category III, but European guidelines are much more conservative in that area [4, 5]. Consequently, multi-gen panels, that are popular in the USA, are used in Europe much less often.

The low agreement of diagnoses of category IV, especially between the centers, was somewhat unexpected. That problem was well shown by the analysis of diagnoses formulated for nodules classified into category IV by pathologist C1: in those cases, pathologists of center B most often diagnosed category III, while pathologist A1 assigned categories IV and V with similar frequency. Pathologist C1 was unique in their attitude to the presence of mild nuclear changes in follicular-patterned cases. The percentage of cancers among nodules diagnosed as FP-NC by pathologist C1 was close to 90% and about 90% of those cancers were PTC, while in the case of other pathologists the FoM in FP-NC cases did not exceed 40%, and the frequency of PTC among those cancers—50%. Those differences resulted in a FoM in category IV nodules three times higher as diagnosed by pathologist C1 than other pathologists. Interestingly, in the case of both pathologists of center B none of the cancers in nodules classified into category IV was PTC, and the majority of them were HTC. Distinguishing HTC cells from PTC cells may be difficult because of similar oxyphilic cytoplasm in both cases. It seems that pathologists of center B avoided assigning smears with cells presenting oncocyte-like cytoplasm and features of PTC into the category IV. It could be a consequence of their habit to use the definition of category IV in its primary form (excluding cases with nuclear features of PTC) in routine diagnostics, according to the national guidelines. On the other hand, pathologist C1 made intensive use of the possibility of assigning cases with mild nuclear changes into that category. That is a good example of how much individual interpretations of Bethesda classification guidelines may differ. Interestingly, in the case of pathologist C1 the FoM in FN-NC nodules was twice as high as the FoM in other nodules of category IV, while in the case of pathologist B1 it was the opposite. That differences were not statistically significant but undoubtedly the impact of FN-NC inclusion into category IV should be analyzed on a higher number of cases and in populations with different iodine supply, which is known to modify the relative incidences of FTC and PTC.

In our material the agreement of diagnoses within the centers was higher than between the centers but still rather unsatisfactory. The higher agreement was probably a consequence of local traditions of thyroid cytology assessment and the cooperation of pathologists during the routine diagnostics. This thesis is confirmed by a relatively high agreement between pathologists B1 and C1 (with the exception of category IV), who despite working in different centers used to consult difficult cases between themselves. The significance of the group consensus in lowering interobserver disagreement in the case of smears with equivocal microscopic image was also indicated by others [16, 17].

Previous studies on the agreement of diagnoses in the Bethesda classification between several pathologists were usually conducted on smaller groups of nodules comprising all categories of the classification. In such a setting the agreement was satisfactory. But when the unequivocal categories (categories II and VI) were excluded from the analysis the agreement decreased [18, 19]. Kocjan et al. also reported poor agreement for categories Thy3a and Thy4 of the UK Royal College of Pathologists’ classification system (equivalent to BSRTC III and V, respectively) [20]. Similar conclusions were drawn by Padmanabhan et al. [21] and Bhasin et al. [22] in the case of category III of the Bethesda classification and Cochand-Priollet et al. [17] in the case of categories III and IV. Our study is special not only for the assessment of the agreement of diagnoses of equivocal categories in a large set of nodules but also for the analysis of the frequency of their malignancy based on postoperative histopathological examination. We used the term ‘FoM’ instead of RoM on purpose. The evaluation of RoM would demand an examination of subsequent nodules with equivocal cytology and known definite diagnosis. Our sample was characterized with the overrepresentation of cancers in the relation to their incidence in the general population that had been exposed to iodine deficiency for a long time. Such a selection of nodules allowed us to address the investigated problem better, but made it impossible to draw any direct conclusions about the RoM of particular BSRTC categories. Such a selection of nodules, as well as the pathologists being aware of it, could be a potential source of bias. Another limitation of our study is the lack of the reassessment of postoperative histopathological examinations. Thus, the actual incidence of NIFTP and other borderline tumors could not be determined.

Despite these limitations, our results indicate a necessity for future studies focused on the standardization of the rules for the classification of smears into particular BSRTC categories, especially equivocal ones. At present, the use of published data on the RoM of nodules in particular BSRTC categories without a concern for the uniqueness of the diagnostic center may lead to erroneous conclusions. That uniqueness is not only a result of specific epidemiological conditions (e.g., iodine supply) but also of variations in the interpretation of diagnostic criteria for each BSRTC category. That interpretation may be modified by a profile of patients who are routinely diagnosed— the profile that is different in oncological and endocrinological centers.

## Supplementary information

Supplementary Information

## Data Availability

(Data transparency)—‘Not applicable’.

## Code availability

(Software application or custom code)—‘Not applicable’.
