# Supplementary material for: Low reproducibility of equivocal categories of the Bethesda System for Reporting Thyroid Cytology makes the associated risk of malignancy specific to the diagnostic center
Source: Endocrine. 2021 Jun 12;74(2):355–64. doi: 10.1007/s12020-021-02781-3 (PMC8497291; doi:10.1007/s12020-021-02781-3)
Supplement: Supplementary file 1 — Supplementary Information [file 12020_2021_2781_MOESM1_ESM.pdf]

Supplementary information

Endocrine

Low reproducibility of equivocal categories of the Bethesda System for Reporting Thyroid Cytology makes the associated risk of malignancy specific to the diagnostic center by Dorota Słowińska-Klencka, Mariusz Klencki, Joanna Duda-Szymańska, Jarosław Szwalski, Bożena Popowicz

Corresponding author:

Dorota Słowińska-Klencka
Department of Morphometry of Endocrine Glands, Chair of Endocrinology, Medical University of Lodz, Pomorska Str 251, 92-213, Łódź, Poland;
e-mail – dsk@tyreo.umed.lodz.pl phone – (48) 42-201-44-14

Supplementary Table 1. The distribution of categories of FNA outcomes by all pathologists in the cases classified into category III of the Bethesda classification by one of them.

category III No of cases	other pathologists	categories of the Bethesda classification - No/% of cases					
		I	II	III	IV	V	VI
**A1: 68**	A2		4/5.9	47/69.1	11/16.2	6/8.8	
	B1	7/10.3	14/10.6	46/67.6		1/1.5	
	B2	1/1.5	12/17.7	52/76.5	3/4.4		
	C1	4/5.9	14/20.6	47/69.1	2/2.9	1/1.5	
**A2: 76**	A1			47/61.8	18/23.7	11/14.5	
	B1	4/5.3	14/18.4	56/73.7	2/2.6		
	B2	1/1.3	10/13.2	59/77.6	2/2.6	4/5.2	
	C1	4/5.3	18/23.7	50/65.8	4/5.3		
**B1: 157**	A1			46/29.3	65/41.4	42/28.7	1/0.6
	A2		2/1.3	56/35.7	69/43.9	27/17.2	3/1.9
	B2		7/4.4	119/75.8	18/11.5	12/8.3	
	C1	1/0.6	12/7.6	131/83.4	11/7.0	1/0.6	1/0.6
**B2: 136**	A1			52/38.2	48/35.3	36/26.5	
	A2		2/1.5	59/43.4	51/37.5	23/16.9	1/0.7
	B1	4/2.9	11/8.1	119/87.5	2/1.5		
	C1	2/1.5	18/13.2	104/76.5	9/6.6	1/0.7	2/1.5
**C1: 152**	A1			47/30.9	66/43.4	39/25.7	
	A2		4/2.6	50/32.9	71/46.7	24/15.8	3/2.0
	B1	3/2.0	8/5.3	131/86.2	6/3.9	4/2.6	
	B2		11/7.2	104/68.4	22/14.5	15/9.9	

Supplementary Table 2. The distribution of categories of FNA outcomes by all pathologists in the cases classified into category IV of the Bethesda classification by one of them.

**category IV No of cases**	**other pathologists**	**categories of the Bethesda classification - No/% of cases**					
		**I**	**II**	**III**	**IV**	**V**	**VI**
**A1: 79**	A2		1/1.3	18/22.8	59/74.7	1/1.3	
	B1		4/5.1	65/82.3	9/11.4	1/1.3	
	B2		4/5.1	48/60.8	24/30.4	3/3.8	
	C1		5/6.3	66/83.5	8/10.1		
**A2: 81**	A1			11/13.6	59/72.8	11/13.6	
	B1		3/3.7	69/85.2	7/8.6	2/2.5	
	B2		2/2.5	51/63.0	22/27.2	6/7.4	
	C1		3/3.7	71/87.7	7/8.6		
**B1: 9**	A1				9/100.0		
	A2			2/22.2	7/77.8		
	B2				9/100.0		
	C1			6/66.7	3/33.3		
**B2: 27**	A1				24/88.9	3/11.1	
	A2			2/7.4	22/81.5	2/7.4	1/3.7
	B1			18/66.7	9/33.3		
	C1			22/81.5	5/18.5		
**C1: 18**	A1			2/11.1	8/44.4	8/44.4	
	A2			4/22.2	7/38.9	4/22.2	3/16.7
	B1		1/5.6	11/61.1	3/16.7	3/16.7	
	B2			9/50.0	5/27.8	3/16.7	1/5.6

Supplementary Table 3. The distribution of categories of FNA outcomes by all pathologists in the cases classified into category V of the Bethesda classification by one of them.

**category V No of cases**	**other pathologists**	**categories of the Bethesda classification - No/% of cases**					
		**I**	**II**	**III**	**IV**	**V**	**VI**
**A1: 62**	A2			11/17.7	11/17.7	31/50.0	9/14.4
	B1		2/3.2	45/72.6		12/19.4	3/4.8
	B2			36/58.1	3/4.8	18/29.0	5/8.0
	C1		5/8.1	39/62.9	8/12.9	4/6.5	6/9.7
**A2: 39**	A1			6/15.4	1/2.6	31/79.5	1/2.6
	B1	2/5.1	1/2.6	27/69.2	-	9/23.1	
	B2		1/2.5	23/59.0	2/5.1	12/30.8	1/2.6
	C1		2/5.1	24/61.5	4/10.3	5/12.8	4/10.3
**B1: 14**	A1			1/7.1	1/7.1	12/85.7	
	A2				2/14.3	9/64.3	3/21.4
	B2			2/14.3		10/71.4	2/14.3
	C1			4/28.6	3/21.4	4/28.6	3/21.4
**B2: 25**	A1			3/12.0	3/12.0	18/72.0	1/4.0
	A2			4/16.0	6/40.0	12/48.0	3/12.0
	B1	1/4.0	1/4.0	13/52.0		10/40.0	
	C1	1/4.0	1/4.0	15/60.0	3/12.0	3/12.0	2/8.0
**C1: 5**	A1			1/20.0		4/80.0	
	A2					5/100.0	
	B1			1/20.0		4/80.0	
	B2			1/20.0		3/60.0	1/20
